# Biodiverse *Histoplasma* Species Elicit Distinct Patterns of Pulmonary Inflammation following Sublethal Infection

**DOI:** 10.1128/mSphere.00742-20

**Published:** 2020-08-26

**Authors:** Grant S. Jones, Victoria E. Sepúlveda, William E. Goldman

**Affiliations:** a Department of Microbiology and Immunology, University of North Carolina at Chapel Hill, Chapel Hill, North Carolina, USA; University of Georgia

**Keywords:** *Histoplasma*, alveolar macrophages, histoplasmosis, inflammation, lung infection, respiratory pathogens, species diversity

## Abstract

*Histoplasma* is an endemic dimorphic fungus that can cause disease in healthy and immunocompromised individuals after the transition of inhaled spores into the facultative intracellular yeast form. There is substantial diversity among *Histoplasma* species, but it is not clear how this heterogeneity impacts the progression of pathology and cellular immune responses during acute respiratory infection, which represents the vast majority of histoplasmosis disease burden. After inoculating mice intranasally with a sublethal inoculum, we characterized the immune response to Histoplasma capsulatum (strain G186A) and Histoplasma ohiense (strain G217B) using comprehensive flow cytometric and single-cell analyses. Within 8 days after inoculation, H. ohiense induced a significantly higher infiltration of neutrophils and inflammatory monocytes into the lung compared to H. capsulatum. Microscopic analysis of infected lung tissue revealed that although the total number of fungi was similar within inflamed lung lesions, we observed different species-dependent intracellular yeast distribution patterns. Inoculation with *gfp*-expressing strains indicated that H. ohiense, but not H. capsulatum, was associated primarily with alveolar macrophages early after infection. Interestingly, we observed a significant reduction in the total number of alveolar macrophages 12 to 16 days after *H. ohiense,* but not H. capsulatum infection, despite similar intracellular growth dynamics within AMJ2-C11 alveolar macrophages *in vitro*. Together, our data suggest that *H. ohiense*, but not H. capsulatum, preferentially interacts with alveolar macrophages early after infection, which may lead to a different course of inflammation and resolution despite similar rates of fungal clearance.

**IMPORTANCE** Acute pulmonary histoplasmosis in healthy individuals comprises most of the disease burden caused by the fungal pathogen *Histoplasma*. Fungal pneumonia is frequently delayed in diagnosis and treatment due to a prolonged period of quiescence early during infection. In this study, we used a murine respiratory model of histoplasmosis to investigate how different *Histoplasma* species modulate lung inflammation throughout the complete course of infection. We propose that a relatively low, sublethal inoculum is ideal to model acute pulmonary histoplasmosis in humans, primarily due to the quiescent stage of fungal growth that occurs in the lungs of mice prior to the initiation of inflammation. Our results reveal the unique course of lung immunity associated with divergent species of *Histoplasma* and imply that the progression of clinical disease is considerably more heterogeneous than previously recognized.

## Abstract (precis)

Acute pulmonary histoplasmosis in healthy individuals comprises most of the disease burden caused by the fungal pathogen *Histoplasma*. Fungal pneumonia is frequently delayed in diagnosis and treatment due to a prolonged period of quiescence early during infection. In this study, we used a murine respiratory model of histoplasmosis to investigate how different *Histoplasma* species modulate lung inflammation throughout the complete course of infection. We propose that a relatively low, sublethal inoculum is ideal to model acute pulmonary histoplasmosis in humans, primarily due to the quiescent stage of fungal growth that occurs in the lungs of mice prior to the initiation of inflammation. Our results reveal the unique course of lung immunity associated with divergent species of *Histoplasma* and imply that the progression of clinical disease is considerably more heterogeneous than previously recognized.

## INTRODUCTION

*Histoplasma* species are thermally dimorphic fungi that are extremely diverse in geographical distribution and genetic composition. In the United States, *Histoplasma* is one of three pathogenic fungi that cause endemic mycoses along with *Blastomyces* and *Coccidioides* due to its association with a specific ecological niche and ability to cause infection in immunocompetent individuals (1). Recent evidence suggests that the known range of *Histoplasma* endemicity around the Ohio and Mississippi River valleys is shifting (2), and this is supported by severe histoplasmosis cases that have been reported in nonendemic areas without known travel to the defined endemic region described 50 years ago (3, 4). It is estimated that approximately three million infections occur per year in the United States (5), and yet, surveillance data may reflect only a small fraction of the total disease burden while also underestimating the most severe cases (6). Outside of the United States, *Histoplasma* is endemic in southern Mexico, Central and South America, India, China, and Southeast Asia (7). AIDS-associated histoplasmosis cases continue to reveal previously unrecognized endemic regions (7), and recent estimates suggest that *Histoplasma* causes a higher number of Latin American HIV-associated deaths than tuberculosis (8). Hence, a significant proportion of the human population worldwide is regularly exposed to *Histoplasma* in which potential disease can range from acute respiratory infections in immunocompetent individuals to invasive disseminated histoplasmosis in immunosuppressed patients.

The majority (>90%) of disease caused by *Histoplasma* is thought to be self-limiting acute pulmonary histoplasmosis. This form of disease is often misdiagnosed for community-acquired viral or bacterial pneumonia (5), which might be at least partially due to low histoplasmosis-specific testing rates, and thus, diagnostic delays can occur after inappropriate antibacterial prescriptions (9). Acute pulmonary illness due to infection with *Histoplasma* is characterized by cough, fever, and chills, and mild cases normally resolve within 1 to 2 weeks untreated, but more severe cases may require up to 12 weeks of antifungal treatment (5). Additionally, it is not uncommon for pulmonary histoplasmosis to be initially a quiescent infection diagnosed incidentally by either biopsy or chest radiograph after the identification of lung nodules or lymphadenopathy (10). An immunocompromised host status is a prerequisite for many fungal infections; however, a recent publication by the CDC found that 80% of all histoplasmosis patients included in their study cohort between 2012 and 2014 were not associated with any immunocompromising condition (9). It is well established that acute pulmonary infection comprises the bulk of human histoplasmosis cases; however, relatively little is known about the progression of lung immunity that leads to fungal clearance following acute infection in mice.

Likewise, even less is known about how the progression of infection is impacted by which *Histoplasma* species was the cause of disease. The wide geographical distribution of histoplasmosis parallels the genetic diversity that exists among *Histoplasma* species. A hallmark publication by Kasuga et al. described seven genetically isolated clades of *Histoplasma* based on genetic variation across four protein-coding genes (11). Teixeria et al. (12) identified additional phylogenetic species of Histoplasma capsulatum within the Latin American clade (now considered Histoplasma suramericanum), and these were proposed to be a result of bat radiation and diversification (12, 13). Whole-genome sequencing, population genetics, and phylogenetic analyses led to the proposal that the previously described H. capsulatum clades represent at least four different genetically isolated species of *Histoplasma* that required taxonomical rearrangement of the genus (13). The composition of the fungal cell wall was the one of the earliest characteristics used to distinguish between *Histoplasma* isolates, which led to the chemotype I (lacking α-glucan) and chemotype II (containing α-glucan) distinctions (14). Most *Histoplasma* strains (including H. capsulatum *sensu stricto*) are chemotype II strains, which require α-(1,3)-glucan for virulence (15); however, virulence is not attenuated in chemotype I strains (H. ohiense, previously referred to as the North American 2 clade) that lack α-(1,3)-glucan in the outer cell wall. Additionally, H. capsulatum and *H. ohiense* have different requirements for virulence determinants, including adhesins, iron homeostasis, and secreted factors (16). Host cell signaling events induced subsequently by various innate immune cell types are dictated by the differential recognition of H. capsulatum and *H. ohiense* yeast cell walls (17).

Much of what is known about *Histoplasma* pathogenesis can be attributed to the use of the G217B isolate of *H. ohiense* combined with high inocula in mice. Historically, the *H. ohiense* G217B isolate is considered more virulent than the H. capsulatum G186A isolate due to higher tissue burdens and more robust inflammation observed in mice after either intravenous or intranasal inoculation with at least 10^5^ CFU (18–20). However, using a low, sublethal intranasal inoculum of 2,500 yeast cells (hereafter referred to as yeasts), we found that H. capsulatum and *H. ohiense* exhibit nearly identical growth kinetics in the lungs of mice during both the acute and resolution stages of infection (19). The intranasal inoculation of mice with 10^3^ spores resulted in 94.1% of mice having positive organ cultures over a 6-month period (21). This suggests that approximately 10^3^ units of infectious material is sufficient to consistently cause disease in mice using a respiratory route of inoculation. Intratracheal inoculation with either 10^3^ H. suramericanum (formerly *Histoplasma* clade LAm A) or *H. ohiense* yeasts led to fungal growth in the lung between 3 and 14 days after inoculation, which was followed by decreased lung burdens 28 days after infection (22). Durkin et al. showed that although total lung burdens were similar between two distinct classes of *Histoplasma*, overall mortality and lung histopathology results suggest diverse host inflammatory responses (22). Therefore, in the context of similar lung fungal burdens, we propose that the use of a relatively low inoculum can provide insight into the distinct pulmonary immune responses induced by diverse *Histoplasma* species. In this study, we characterized unique inflammatory dynamics that were correlated with the two most commonly used *Histoplasma* isolates, H. capsulatum G186A and *H. ohiense* G217B, during the acute and resolution stages of lung infection in immunocompetent mice. Our findings highlight the differential progression of lung inflammation and intracellular parasitism associated with diverse species of *Histoplasma* and could be informative to our understanding of the variety of disease that occurs worldwide.

## RESULTS

### H. capsulatum induces delayed, but prolonged, lung inflammation relative to *H. ohiense* following sublethal inoculation.

**FIG 1:**
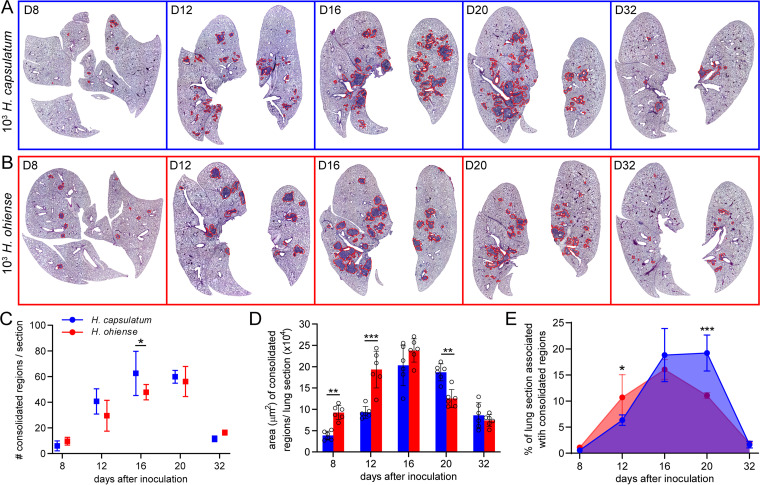
H. capsulatum induces delayed, but prolonged, lung inflammation relative to *H. ohiense* following sublethal inoculation. C57BL/6J mice were inoculated intranasally with a low, sublethal dose (2,500 yeasts) of either H. capsulatum or *H. ohiense*, and lungs were collected at the indicated time points (days) after infection. (A and B) Representative H&E-stained lung image composites (see Fig. S1) from mice infected with either H. capsulatum (blue) or *H. ohiense* (red). Regions outlined in red correspond to inflammatory foci associated with fully consolidated airways. (C) Mean number (± standard deviation [SD] [error bars]) of consolidated regions in each lung section. (D) Bars indicate the mean surface area of individual foci in each lung section. (E) Mean percent (±SD) of total lung area associated with inflammatory foci at each time point. Three lung sections separated by 200-μm skips were analyzed from two mice at each time point for each strain. Statistical significance was determined using unpaired *t* tests and indicated by bars and asterisks as follows: *, *P* < 0.05; **, *P* < 0.01; ***, *P* < 0.001.

To quantify the progression of inflammation during sublethal, acute infection with divergent species of *Histoplasma*, inflamed lesions were analyzed in hematoxylin and eosin (H&E)-stained lung sections (see Fig. S1 in the supplemental material) after intranasal inoculation with 2,500 yeasts of either H. capsulatum (Fig. 1A) or *H. ohiense* (Fig. 1B). During sublethal histoplasmosis in mice, inflammatory foci were not evident until at least 8 days after inoculation, despite this time point being associated with the highest fungal burden during infection (19). Inflammatory foci were most abundant between 16 and 20 days after inoculation with either H. capsulatum or *H. ohiense* (Fig. 1C). The proportion of the total lung area associated with inflamed lesions was consistent with our previous qualitative findings (19), and these foci were primarily centered around medium to large airways and blood vessels. A similar total number of individual regions were quantified in the lungs of mice infected with either *Histoplasma* species 8 days after inoculation (Fig. 1C), whereas the average surface area of each region in the lungs of *H. ohiense*-infected mice was significantly higher than those of H. capsulatum-infected mice (Fig. 1D). Approximately 10% of the lung area was comprised of airways that were completely consolidated 8 days after inoculation with *H. ohiense*, and this was significantly higher than that observed after inoculation with H. capsulatum (Fig. 1E). Later during infection, we observed that the host inflammatory response to H. capsulatum was prolonged relative to *H. ohiense*-triggered inflammation, which had begun to resolve at 16 days postinoculation (Fig. 1E). This was due to the mean size, but not number, of inflammatory lesions that decreased from 16 to 20 days after inoculation with *H. ohiense*, but not H. capsulatum (Fig. 1D). Overall, quantitative image analyses of whole lung sections revealed that H. capsulatum and *H. ohiense* elicited distinct patterns of lung inflammation with regard to the kinetics of airway consolidation and disease resolution.

10.1128/mSphere.00742-20.1 FIG S1 Description of quantitative histopathology. Visual representation and description of the process used to analyze whole-lung images shown in Fig. 1A to evaluate the progression of lung inflammation during *Histoplasma* infection. The representative section shown corresponds to day 16 after inoculation with H. capsulatum (Fig. 1A). Download FIG S1, TIF file, 2.1 MB. Copyright © 2020 Jones et al. 2020 Jones et al. This content is distributed under the terms of the Creative Commons Attribution 4.0 International license.

### Host response to *H. ohiense*, but not H. capsulatum, is associated with a significant influx of neutrophils and a concomitant depletion of alveolar macrophages.

Next, flow cytometry was used to determine the identity of host cells that infiltrated the lung during low-dose, sublethal acute infection with each *Histoplasma* species. Traditional sequential gating was combined with unbiased analyses to identify all CD45^+^ leukocyte populations. This allowed us to determine how leukocyte populations differed in number and phenotype during histoplasmosis. A panel of surface antigens was implemented to phenotype myeloid cell subsets in addition to general lymphoid cell types following enzymatic digestion of lung tissue (23, 24). Live CD45^+^ singlets from all mice at each time point were concatenated and subjected to *t*-distributed stochastic neighbor embedding (tSNE) (unbiased clustering) analysis in FlowJo (Fig. S2A). To determine the identity of distinct cell populations segregated by tSNE, we used a sequential gating scheme (Fig. S2B) to characterize lung immune cell populations (23, 24). Clustered populations were confirmed by antigen expression heat maps generated by the unbiased tSNE algorithm (Fig. S2C). The distribution and abundance of lung cell populations in each group of mice could be directly compared after backgating using the embedded file keywords (Fig. S2A).

10.1128/mSphere.00742-20.2 FIG S2 Combination of sequential gating scheme and tSNE to confirm the phenotype of uniquely clustered immune cell populations in the lung. (A) Workflow in FlowJo used to generate tSNE plots and to ultimately identify species-specific differences in lung immune cell dynamics. (B) Traditional gating scheme used to identify all CD45^+^ lung immune cell populations in a sequential manner. (C) Example heat maps depicting the expression profile of individual surface antigens on populations identified by tSNE using lung cells isolated 8 days after inoculation. Download FIG S2, TIF file, 2.7 MB. Copyright © 2020 Jones et al. 2020 Jones et al. This content is distributed under the terms of the Creative Commons Attribution 4.0 International license.

**FIG 2:**
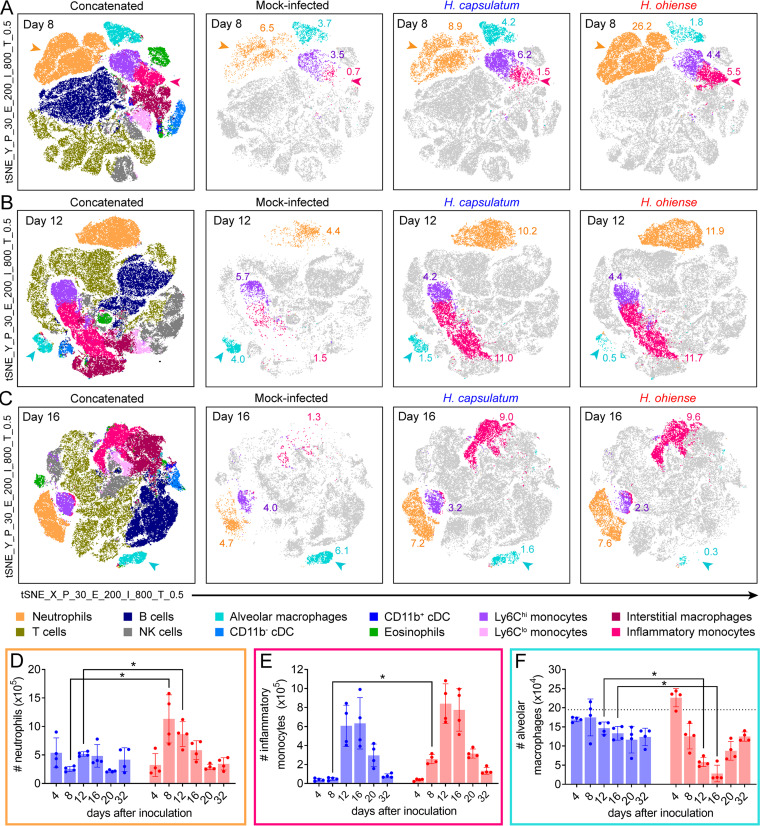
Host response to *H. ohiense*, but not H. capsulatum, is associated with a significant influx of neutrophils and a concomitant depletion of alveolar macrophages. Lung cells from C57BL/6J mice were analyzed at the indicated times after intranasal inoculation with 2,500 H. capsulatum or *H. ohiense*. (A to C) Concatenated live, CD45^+^ lung cells were visualized 8 days (A), 12 days (B), or 16 days (C) after *Histoplasma* infection compared to control mice using a combination of tSNE (see Fig. S2A in the supplemental material) and a sequential gating scheme (Fig. S2B). Concatenated events from mice at each time point depict color-coded cell populations derived from the sequential gating scheme and tSNE expression profiles (Fig. S2C) that were overlaid onto each tSNE plot. Plots to the right include events that were derived from either mock-infected, H. capsulatum-, or *H. ohiense*-inoculated mice, and populations of interest include neutrophils (orange), Ly6C^hi^ MHC-II^−^ monocytes (purple), Ly6C^hi^ MHC-II^+^ inflammatory monocytes (magenta), and alveolar macrophages (cyan). Numbers associated with colored clusters indicate the average percentage among CD45^+^ cells. Each plot includes 20,000 to 40,000 pooled events (10,000 events per mouse with two mock-infected mice per time point or four *Histoplasma*-infected mice per time point). cDC, conventional dendritic cells. (D to F) Bars represent the mean total number (±SD) of neutrophils (D), inflammatory monocytes (E), and alveolar macrophages (F) recovered from the lungs of mice infected with either H. capsulatum (blue bars) or *H. ohiense* (red bars) (*n* = 4 per time point). Horizontal dotted lines indicate the mean number of cells recovered from mock-infected mice (*n* = 10, 1 or 2 mice were analyzed at each time point). Data are representative of two separate time course experiments, and statistical significance was evaluated using nonparametric Mann-Whitney tests; *, *P* < 0.05.

The comprehensive flow cytometric-based analysis of lung cells described above was performed at various times after inoculation with *Histoplasma*, resulting in specific tSNE display patterns at each time point (Fig. 2). Eight days following inoculation, neutrophils (orange) represented a significant proportion of CD45^+^ cells (26%) in response to *H. ohiense*-infected mice relative to H. capsulatum-infected mice, in which only 9% of CD45^+^ lung cells were neutrophils (Fig. 2A and D). Furthermore, infection with H. capsulatum was associated with only a modest neutrophilic response relative to mock-infected mice (Fig. 2D), despite similar total lung fungal burdens as *H. ohiense* (19). *H. ohiense*-infected lungs contained a significantly higher number of inflammatory monocytes (magenta) relative to H. capsulatum infection, despite a similar proportion of naive Ly6C^hi^ monocytes (purple) at this time (Fig. 2A and E). As the infection progressed, the proportion and total number of inflammatory monocytes recruited to the lungs were similar between both *Histoplasma* species (Fig. 2B, C, and E). These results are consistent with our lung histological analyses and suggest that acute inflammation in response to *H. ohiense* was associated with the early recruitment of neutrophils and inflammatory monocytes into the lung.

Despite a similar proportion of naive and inflammatory monocytes 12 to 16 days after inoculation, we observed a significant loss of alveolar macrophages (cyan) in response to *H. ohiense*, but not H. capsulatum (Fig. 2B, C, and F). The total number of alveolar macrophages declined between 4 to 8 days after inoculation with *H. ohiense* and continued to decrease until 16 days postinoculation (Fig. 2F). At this time, the number of alveolar macrophages recovered from the lungs of *H. ohiense*-infected mice was reduced nearly fivefold compared to H. capsulatum (Fig. 2F). However, the number of alveolar macrophages began to rise from 20 to 32 days after infection with *H. ohiense* (Fig. 2F), which correlated with the resolution of inflammation (Fig. 1). Overall, alveolar macrophages were the only CD45^+^ lung cell population that was selectively depleted in response to *H. ohiense* relative to H. capsulatum.

### *In situ* visualization of *Histoplasma* reveals unique intracellular growth dynamics.

**FIG 3:**
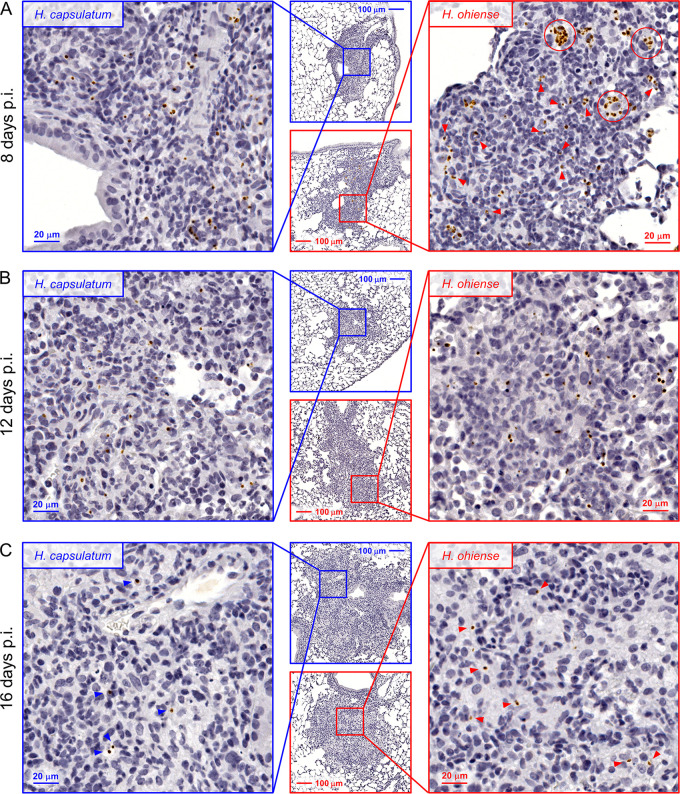
*In situ* visualization of *Histoplasma* reveals unique intracellular growth dynamics. (A to C) Lungs were harvested on day 8 (A), day 12 (B), or day 16 (C) after inoculation with either *gfp*-expressing H. capsulatum or *H. ohiense*. Immunohistochemistry was performed on serial sections to chromogenically detect anti-GFP followed by hematoxylin staining. Red circles in panel A highlight heavily infected mononuclear phagocytes in the lungs of *H. ohiense*-infected mice, and arrowheads point to cells associated with few yeasts per cell. Blue and red arrowheads in panel C highlight yeasts detected within lung inflammatory foci 16 days after inoculation. p.i., postinoculation.

It has been presumed that alveolar macrophages are an important intracellular growth niche for *Histoplasma* during pulmonary infection, and this led us to hypothesize that alveolar macrophages heavily infected with *H. ohiense* may have been destroyed during the generation of single-cell suspensions. Therefore, we opted to use an *in situ* approach to visualize *Histoplasma-*infected cells by inoculating mice with *gfp-*expressing *Histoplasma* and then performing immunohistochemistry to chromogenically label green fluorescent protein (GFP) within yeasts, followed by secondary hematoxylin staining. We used this approach over fluorescence-based techniques to avoid autofluorescence in formalin-fixed lung tissue, to avoid photobleaching of local GFP signal during the time required to scan the whole lung section, and to improve long-term storage stability. Four days after inoculation, we were unable to infer any species-specific trends due to a lack of inflammation and a limited number of infected cells. However, *Histoplasma* yeasts were readily identified in the lungs 8 days after inoculation, and both species were always found within inflammatory foci (Fig. 3A). The majority of H. capsulatum-infected cells in the lung were localized with only one or two yeasts on average, and we rarely visualized single cells associated with more than four or five yeasts (Fig. 3A). In contrast, *H. ohiense-*infected tissue sections contained heavily infected mononuclear phagocyte populations (circled in red in Fig. 3A). The relatively large and rounded nuclei of these cells localized in alveoli were consistent with macrophages and not inflammatory or resident monocytes. Despite the presence of heavily infected cells, most *H. ohiense* yeasts were localized with smaller mononuclear and polymorphonuclear cells (red arrowheads in Fig. 3A). In regards to the general immune infiltrate, polymorphonuclear leukocytes were abundant within the same inflammatory foci of *H. ohiense*-infected lungs, which was consistent with the significantly elevated number of neutrophils relative to H. capsulatum infection (Fig. 2D). The average number of yeasts associated with inflamed lesions was not significantly different between H. capsulatum and *H. ohiense* (Fig. S3). Overall, these microscopy findings 8 days after inoculation suggested that H. capsulatum infected a range of mononuclear cells with distinct morphologies, and these cells contained only a few yeasts per cell on average. In contrast, while several large mononuclear cells were heavily infected with *H. ohiense*, the majority of yeasts were associated with smaller leukocytes (presumably inflammatory granulocytes and monocytes) that had only a few yeasts per cell.

10.1128/mSphere.00742-20.3 FIG S3 The number of lesion-associated yeasts is similar between *Histoplasma* species. Bars indicate the mean number of yeasts associated with inflammatory foci on day 8, day 12, or day 16 after inoculation. Yeasts were identified by chromogenic detection of anti-GFP and secondary hematoxylin staining. The number of yeasts per lesion was quantified relative to the area of each lesion using the counting tool and pen tool, respectively, in ImageScope. Three lung sections separated by 200-μm skips were analyzed from one mouse per time point and were derived from two separate experiments. *P* values were calculated using Mann-Whitney analyses. Download FIG S3, TIF file, 0.1 MB. Copyright © 2020 Jones et al. 2020 Jones et al. This content is distributed under the terms of the Creative Commons Attribution 4.0 International license.

Next, we turned our attention to relatively later stages of infection associated with fungal clearance and the resolution of inflammation. Twelve to 16 days after inoculation, we did not observe more than two or three yeasts of either *Histoplasma* species associated with individual cells (Fig. 3B and C). A similar number of yeasts were enumerated in the inflammatory foci of lungs 12 days after inoculation with either *Histoplasma* species (Fig. S3). Lung tissue examined 16 days after inoculation (Fig. 3C) contained the largest inflamed regions, which was consistent with the quantitative data in Fig. 1. Despite the large size of these inflammatory foci, relatively few yeasts were detected within these regions 16 days after inoculation (Fig. 3C), and again, there was not a significant difference in the number of yeasts quantified between fungal species (Fig. S3). Therefore, these histological findings serve as compelling evidence that the preferential depletion of alveolar macrophages 16 days following *H. ohiense* infection is not a result of heavily infected cells that had lysed during tissue processing. Likewise, as highlighted in Fig. 3C, it was uncommon to identify *H. ohiense*-associated cells that had more than one yeast per cell at this time point. Therefore, the reduction in the number of yeasts within individual consolidated regions directly correlated with the resolution of acute histoplasmosis in mice.

### *H. ohiense* is preferentially associated with alveolar macrophages early after infection.

**FIG 4:**
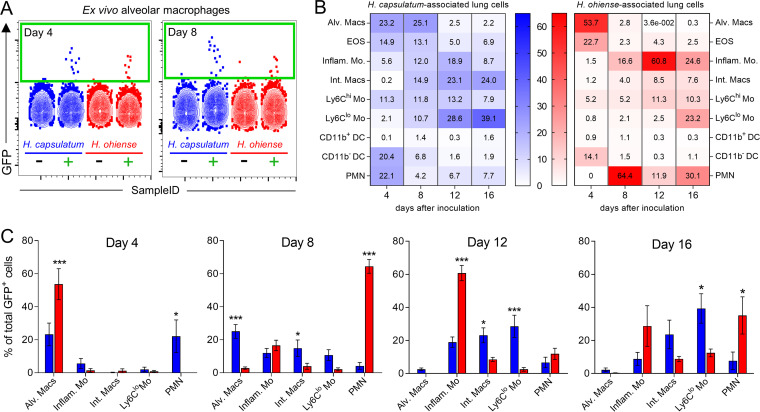
*H. ohiense* is preferentially associated with alveolar macrophages early after infection. Alveolar macrophages (Siglec-F^+^ CD11c^+^ CD64^+^ CD11b^−/+^ CD24^−^) were gated at each time point after inoculation with either *gfp-*expressing H. capsulatum or *H. ohiense*, or nonfluorescent control strains. (A) Representative flow cytometry plots illustrate the detection of *Histoplasma*-associated alveolar macrophages between mice inoculated with control strains of each species (black minus sign) compared to *gfp-*expressing strains (green plus sign) at two different time points. (B) Heat maps show the distribution of *Histoplasma*-associated cells 4, 8, 12, and 16 days after inoculation. Numbers indicate the average proportion of each cell type infected with *Histoplasma* (rows) relative to the total number of GFP^+^ cells at each time point (columns). (C) Bar graphs represent the mean (± standard error of the mean [SEM]) percentage of all GFP^+^ cells associated with each cell type that were significantly different between *Histoplasma* species. Data are pooled from two separate experiments (*n* = 7 per species at each time point). Statistical significance was evaluated between *Histoplasma* species at each time point using unpaired *t* tests; *, *P* < 0.05; **, *P* < 0.01; ***, *P* < 0.001. Abbreviations: Alv. Macs, alveolar macrophages; EOS, eosinophils; Inflam. Mo, inflammatory monocytes; Int. Macs, interstitial macrophages; DC, dendritic cells; PMN, polymorphonuclear leukocyte.

To determine the phenotype of specific leukocyte populations associated with *Histoplasma* in the lung, we inoculated mice intranasally with *gfp-*expressing *Histoplasma*, and flow cytometry was used to identify GFP-positive (GFP^+^) cells. Despite a total of only 10^3^ to 10^4^ GFP^+^ CD45^+^ lung cells 4 to 8 days after infection (Fig. S4), GFP^+^ alveolar macrophages were easily identified using mice inoculated with nonfluorescent control strains as negative gating controls (Fig. 4A). We observed species-dependent differences in the proportion of GFP^+^ cells among myeloid populations at each time point (Fig. 4B and C). Four days after inoculation, approximately 54% of all GFP^+^ cells isolated from the lungs of *H. ohiense*-infected mice were alveolar macrophages, compared to only ∼23% of GFP^+^ cells during infection with H. capsulatum (Fig. 4B and C). The proportion of GFP^+^ alveolar macrophages remained consistent from 4 to 8 days after inoculation with H. capsulatum, whereas less than ∼3% of all GFP^+^ cells were alveolar macrophages 8 days after inoculation with *H. ohiense* (Fig. 4B). The vast majority of GFP^+^ cells were neutrophils 8 days after *H. ohiense* infection (Fig. 4B and C), which correlated with the significant influx of neutrophils into the lungs of *H. ohiense-*infected mice, but not H. capsulatum-infected mice, at this time point (Fig. 2D).

10.1128/mSphere.00742-20.4 FIG S4 Similar total numbers of *Histoplasma*-associated lung cells between fungal species. Average total number of GFP^+^ cells in the lungs of each mouse at various time points after inoculation with either *gfp*-expressing H. capsulatum or *H. ohiense*. Data are pooled from two separate experiments, and each symbol represents the value for an individual mouse. Download FIG S4, TIF file, 0.1 MB. Copyright © 2020 Jones et al. 2020 Jones et al. This content is distributed under the terms of the Creative Commons Attribution 4.0 International license.

Later during infection with both *Histoplasma* species, the distribution of fungus-associated cells shifted toward other mononuclear phagocytes, including monocytes and interstitial macrophages (Fig. 4B). Approximately 60% of all *H. ohiense*-infected cells 12 days after inoculation were Ly6C^hi^ MHC-II^+^ (major histocompatibility complex class II-positive) inflammatory monocytes as opposed to H. capsulatum yeasts, which were localized predominantly with Ly6C^lo^ monocytes and interstitial macrophages (Fig. 4B and C). Fungal clearance in the lung occurred from 16 to 20 days after inoculation with either *Histoplasma* species (19), and accordingly, we detected slightly fewer GFP^+^ cells at this time point relative to 12 days postinoculation (Fig. S4). A significantly higher proportion of total GFP^+^ cells corresponded to Ly6C^lo^ monocytes 16 days after inoculation with H. capsulatum, whereas *H. ohiense* yeasts were mainly associated with inflammatory monocytes and neutrophils at this time (Fig. 4B and C). Together with our histology findings, our data suggest that H. capsulatum does not preferentially localize or replicate inside a specific leukocyte population during the progression of disease. In contrast, *H. ohiense* is primarily associated with alveolar macrophages early after infection before being taken up by neutrophils and inflammatory monocytes that are recruited to the lung during infection. These yeast localization studies reveal unique host cell association patterns that are *Histoplasma* species dependent and further support the distinct progression of lung inflammation and host cell recruitment induced by each fungal species.

### Invasion and intracellular growth of *Histoplasma* in alveolar macrophages is species independent.

To determine whether the preferential association of *H. ohiense* with alveolar macrophages was due to enhanced invasion and intracellular replication relative to H. capsulatum, we investigated the interaction of *Histoplasma* with alveolar macrophages *in vitro.* The transformed alveolar macrophage cell line AM2J-C11 is derived from C57BL/6 alveolar macrophages and functions similarly to the parental population (25). We employed this alveolar macrophage cell line for our single-cell analyses as opposed to *ex vivo* isolation because only ∼75% of bronchoalveolar lavage cells are MHC-II^−^ alveolar macrophages (26). In addition, we wanted to avoid potentially triggering a proinflammatory response that can occur in freshly isolated alveolar macrophages (27).

**FIG 5:**
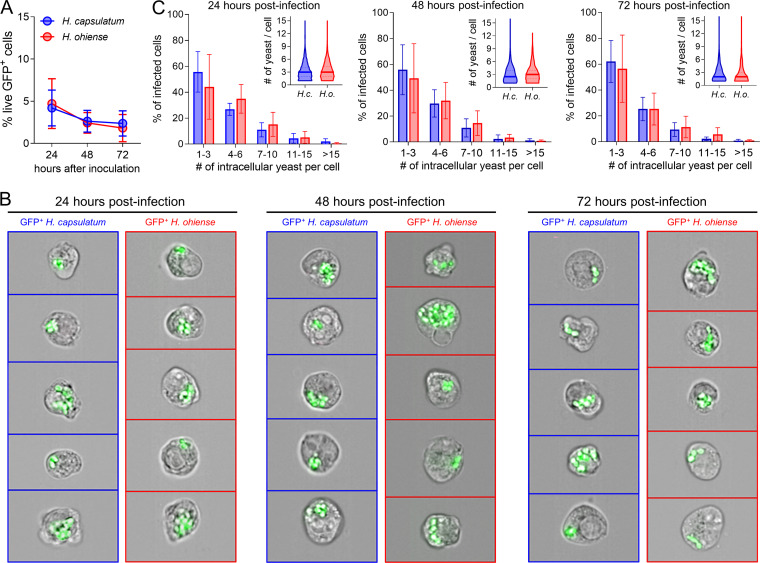
Invasion and intracellular growth of *Histoplasma* in alveolar macrophages is species independent. (A to C) Alveolar macrophages (AMJ2-C11) were inoculated *in vitro* with *gfp*-expressing H. capsulatum (blue) or *H. ohiense* (red) at a ratio of 0.5 to one yeast per cell and analyzed 24 to 72 h later using an imaging flow cytometer. (A) Mean (± SD) percentage of live cells that were GFP^+^ at the indicated time points after *in vitro* infection with either H. capsulatum or *H. ohiense*. (B) Representative images of live, GFP^+^ cells 24 to 72 h after incubation with each *gfp-*expressing *Histoplasma* strain. (C) Bars indicate the mean (± SD) proportion of GFP^+^ cells at each time point that harbored the number of intracellular yeasts denoted on the *x* axis. Violin plots inside each bar graph indicate the number of yeasts per infected cell; solid horizontal lines represent the median values, and dashed lines are quartiles. The number of intracellular yeasts were quantified in an average of 350 GFP^+^ cells per sample, and data were pooled from two separate ImageStream experiments.

To avoid overwhelming cells with an excessive number of yeasts immediately after incubation, AMJ2-C11 cells were incubated with *gfp-*expressing *Histoplasma* yeasts at a low ratio (0.5 to 1 yeast per cell) to monitor invasion and intracellular replication of *Histoplasma*. Imaging flow cytometry approaches have superior sensitivity and accuracy compared to lysing cells and enumerating CFU due to differences in plating efficiency and aggregation of H. capsulatum and *H. ohiense* yeasts (28). Additionally, this single-cell analysis allowed us to visualize individually infected cells in contrast to CFU recovered from the entire population of infected cells. We hypothesized that *H. ohiense* would be taken up more efficiently by alveolar macrophages relative to H. capsulatum. However, similar percentages of AMJ2-C11 cells were associated with H. capsulatum and *H. ohiense* 24 h after infection (Fig. 5A). The percentage of live GFP^+^ cells did not increase from 24 to 72 h after initial infection, indicating a lack of secondary infection that can result from extracellular fungal outgrowth (Fig. 5A). Likewise, the number of dead GFP^+^ cells (identified by uptake of a viability dye) was minimal during *in vitro* infection (Fig. S5A), which was consistent with a previous study using a similar multiplicity of infection in macrophages (28). Therefore, we concluded that *H. ohiense* was not taken up by alveolar macrophages more efficiently than H. capsulatum. These data also suggest that infection with *H. ohiense* does not induce overt cell death relative to H. capsulatum when macrophages are exposed to a relatively low inoculum.

10.1128/mSphere.00742-20.5 FIG S5 Additional data to support *in vitro* infection studies. (A) Mean (±SD) percentage of dead AMJ2-C11 cells (positive for viability dye) that were GFP^+^ at the indicated time points after *in vitro* infection with either H. capsulatum or *H. ohiense* at a ratio of 0.5 to 1 yeast per cell. (B) Growth curves of control and *gfp*-expressing H. capsulatum and *H. ohiense* strains in complete DMEM medium supplemented with l-cystine that was used to culture AMJ2-C11. Download FIG S5, TIF file, 0.2 MB. Copyright © 2020 Jones et al. 2020 Jones et al. This content is distributed under the terms of the Creative Commons Attribution 4.0 International license.

H. capsulatum and *H. ohiense* had similar *in vitro* generation times with logarithmic phase growth occurring between 24 and 72 h in the same media used to culture AMJ2-C11 (Fig. S5B). Additionally, these growth curves were consistent with previous *in vitro* growth curves in standard *Histoplasma* growth media (15). To investigate the intracellular growth dynamics of each *Histoplasma* species, imaging flow cytometry was used to quantify the number of yeasts inside each GFP^+^ AMJ2-C11 cell. Composite images of live, GFP^+^ AMJ2-C11 cells 24 to 72 h after infection were generated by merging images from bright-field and GFP channels (Fig. 5B). Next, the spot count feature was used to enumerate the number of intracellular yeasts within each GFP^+^ cell. At each time point analyzed, most infected cells had six or fewer yeasts per cell (Fig. 5C) with a median of approximately three yeasts per cell regardless of *Histoplasma* species (Fig. 5C, inset). There was not an appreciable increase in the median number of intracellular yeasts per cell from 24 to 72 h after inoculation with either fungal species (Fig. 5C). This was likely due to this approach providing a snapshot of individually infected cells at each time point, instead of tracking growth within the same cell, which would be technically challenging given the slow doubling time of *Histoplasma*. Overall, these *in vitro* studies indicated that H. capsulatum and *H. ohiense* exhibited similar uptake and intracellular growth rates with cultured alveolar macrophages.

### Infection with *H. ohiense* leads to enhanced alveolar macrophage activation relative to H. capsulatum.

**FIG 6:**
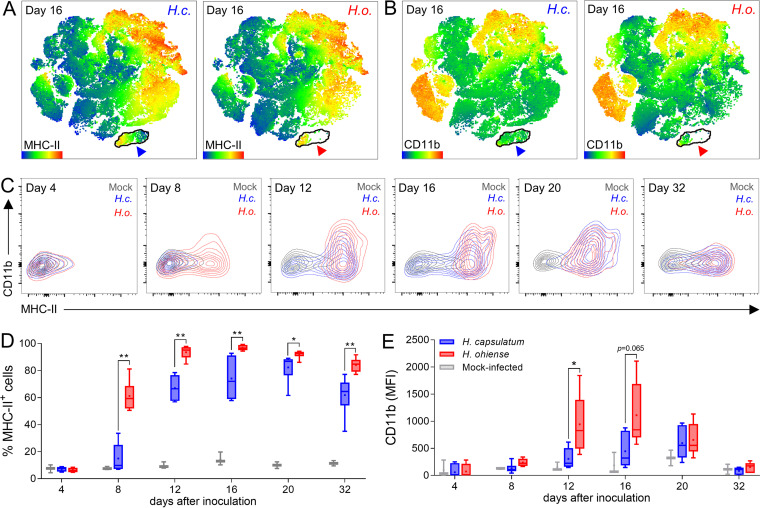
Infection with *H. ohiense* leads to enhanced alveolar macrophage activation relative to H. capsulatum. (A and B) tSNE heat maps (generated as in Fig. S2B) depict the intensity of MHC-II expression (A) or CD11b expression (B) on lung cells 16 days after inoculation with either H. capsulatum (*H.c.*) or *H. ohiense* (*H.o*.). Alveolar macrophages are outlined in black, and the arrowhead color corresponds to cells that were derived from the lungs of mice infected with either H. capsulatum (blue) or *H. ohiense* (red). (C to E) Alveolar macrophages (Siglec-F^+^ CD11c^+^ CD64^+^ CD11b^−/+^ CD24^−^) were gated at each time point after inoculation with either H. capsulatum (blue), *H. ohiense* (red), or media alone (gray). (C) Representative bivariate contour plots depict the changes in expression of MHC-II and CD11b on alveolar macrophages during the course of infection with either H. capsulatum (*H.c*.) or *H. ohiense* (*H.o*.) compared to control mice. (D and E) Box and whisker plots indicate the percentage of MHC-II^+^ alveolar macrophages (D) and fluorescence intensity (mean fluorescence intensity [MFI]) of CD11b expression (E) at the denoted time points after inoculation (pooled data, *n* = 6 mice for each species at each time point). Statistical significance was evaluated between *Histoplasma* species at each time point using nonparametric Mann-Whitney tests; *, *P* < 0.05; **, *P* < 0.01.

Although we observed that alveolar macrophages were significantly depleted during *H. ohiense* infection, the reasons for this were not immediately apparent. It was unlikely that this could be attributed primarily to yeast-mediated killing, since flow cytometry and histology showed that only a tiny fraction of the alveolar macrophage population was infected. Additionally, *in vitro* growth assays revealed that *H. ohiense* did not replicate more efficiently inside alveolar macrophages relative to H. capsulatum. Therefore, we shifted our focus back to defining parameters of the differential host response against *Histoplasma*. Upon closer observation of the alveolar macrophage populations clustered by tSNE, we noticed that the remaining alveolar macrophages in the lungs of *H. ohiense*-infected mice were localized to only a partial region of the area that corresponded to alveolar macrophages recovered from either mock-infected or H. capsulatum-infected mice 16 days after inoculation (Fig. 2C, cyan arrowheads). This suggested that the remaining alveolar macrophages in the lungs of *H. ohiense*-infected mice expressed a phenotype representative of only a fraction of the alveolar macrophages recovered from either H. capsulatum-infected or mock-infected mice. Alveolar macrophage activation has been associated with elevated levels of MHC-II and CD11b in mice and humans (29). Accordingly, the individual tSNE antigen heat maps revealed that alveolar macrophages isolated from *H. ohiense*-infected lungs had higher relative MHC-II levels (Fig. 6A, light green and yellow events) in addition to elevated levels of CD11b (Fig. 6B, light green events). Bivariate contour plots of CD11b and MHC-II expression on alveolar macrophages revealed the differential rate of cellular activation during infection with divergent species of *Histoplasma* (Fig. 6C). Despite similar total numbers of alveolar macrophages in the lung, over half of the population was MHC-II^+^ by 8 days postinoculation with *H. ohiense* (Fig. 6D). Nearly the entire population of alveolar macrophages in the lungs of *H. ohiense*-infected mice was MHC-II^+^ 12 to 16 days after infection (Fig. 6D). Likewise, CD11b expression levels were significantly higher on alveolar macrophages collected from the lungs of *H. ohiense-* but not H. capsulatum-infected mice 12 and 16 days after infection (Fig. 6E). These time points corresponded to when significantly fewer total alveolar macrophages were recovered from the lungs of *H. ohiense*-infected mice relative to infection with H. capsulatum (Fig. 2F). Together, our data suggest that the relatively few remaining alveolar macrophages in the lungs of *H. ohiense*-infected mice are immunologically distinct from those isolated from H. capsulatum-infected mice. These findings support the idea that infection with *H. ohiense* triggers elevated proinflammatory responses relative to H. capsulatum that modulate the alveolar macrophage population and perhaps the polarization of other mononuclear phagocytes.

### Lung inflammatory mediators induced by *H. ohiense* correlate with immune cell dynamics.

**FIG 7:**
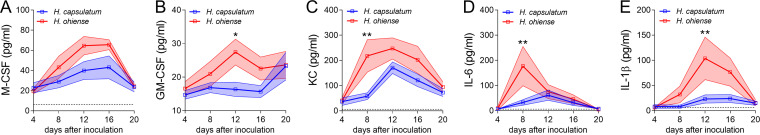
Lung inflammatory mediators induced by *H. ohiense* correlate with immune cell dynamics. (A to E) Mean (±SD) concentrations of myeloid-associated stem cell and inflammatory cytokines detected in whole-lung homogenates at various time points after inoculation with either H. capsulatum or *H. ohiense* yeasts. Shaded area corresponds to the range of the standard error of the mean within groups of mice pooled from two separate infections (*n* = 7 per strain at each time point). The limits of detection are denoted by horizontal dashed lines, and statistical significance was evaluated between *Histoplasma* species at each time point using unpaired *t* tests; *, *P* < 0.05; **, *P* < 0.01.

Last, we sought to determine whether the differential course of leukocyte population dynamics in the lung correlated with myeloid-associated inflammatory mediators. First, we focused on stem cell factors responsible for the survival, recruitment, and proliferation of alveolar macrophages. Macrophage colony-stimulating factor (M-CSF) is important for the establishment and survival of F4/80^+^ resident mononuclear phagocytes in a local manner (30), and granulocyte-macrophage colony-stimulating factor (GM-CSF) is crucial to maintain pulmonary homeostasis by regulating alveolar macrophage growth and differentiation (31, 32). This led us to hypothesize that significantly reduced M-CSF and GM-CSF concentrations during infection with *H. ohiense* were responsible for the depletion of alveolar macrophages. In contrast, neither M-CSF nor GM-CSF levels were significantly reduced in the lungs of mice infected with *H. ohiense* relative to H. capsulatum (Fig. 7A and B). In fact, we observed significantly higher GM-CSF lung concentrations 12 days after inoculation with *H. ohiense* relative to H. capsulatum (Fig. 7B). GM-CSF has been shown to be elevated during inflammatory conditions (33), and this was consistent with the significantly higher number of inflammatory monocytes and neutrophils recruited to the lung relatively early after *H. ohiense*, but not H. capsulatum, infection (Fig. 2D). These data suggest that either local concentrations of stem cell factors are insufficient to support alveolar macrophage survival or growth or that other characteristics of the host response to *H. ohiense* contribute to the depletion of alveolar macrophages.

One of the primary murine chemokines responsible for attracting neutrophils is keratinocyte-derived chemokine (KC) (CXCL1) (34). As expected, we detected significantly higher KC concentrations in the lungs of *H. ohiense*-infected mice relative to inoculation with H. capsulatum (Fig. 7C). This directly correlated with the significantly higher number of neutrophils that infiltrated the lungs 8 days after inoculation with *H. ohiense*, but not H. capsulatum (Fig. 2D). In addition, *H. ohiense* induced elevated concentrations of interleukin-6 (IL-6) that peaked 8 days after infection (Fig. 7D), which closely mirrored the expansion and contraction of neutrophils (Fig. 7D). These results were consistent with a model proposing that IL-6 functions as a regulator of acute inflammation by reducing neutrophil recruitment and promoting the subsequent infiltration of monocytes to help resolve inflammation (35). Interleukin-1 signaling and IL-1β are known to be critical for defense against pulmonary *Histoplasma* infection in mice (36), and previously, we found that *H. ohiense* induced significantly higher IL-1β levels than H. capsulatum 8 days after inoculation with 10^5^ yeasts (19). However, it was unclear whether a low, sublethal inoculum would elicit the same host response pattern between *Histoplasma* species in the context of matched lung burdens. As shown in Fig. 7E, significantly higher IL-1β levels were detected in the lungs of *H. ohiense*-infected mice than in the lungs of H. capsulatum-infected mice, particularly at 12 days postinoculation, which was inversely correlated with the depletion of alveolar macrophages. These findings support our histopathology analyses and quantitation of inflammatory monocytes, which subsequently peaked at 16 days after inoculation, presumably in response to soluble inflammatory factors in the lung.

Overall, our results implied that biodiverse *Histoplasma* species induced distinct patterns of lung inflammation which correlated with the differential composition of innate immune cells and soluble mediators. This underscores the potentially diverse clinical outcomes of pulmonary histoplasmosis that might occur with phylogenetically distinct *Histoplasma* species.

## DISCUSSION

In this study, we identified unique patterns of lung immune responses elicited by geographically and genetically distinct *Histoplasma* species using a murine model of pulmonary disease. Previously, we reported that the inoculation of immunocompetent mice with a low, sublethal dose of various *Histoplasma* species resulted in similar fungal growth and clearance kinetics in the lung (19). Here, we showed that despite a similar number of infected lung cells, H. capsulatum and *H. ohiense* were associated with distinct intracellular niches in the lung. In addition, infection with *H. ohiense* resulted in an enhanced infiltration of neutrophils and inflammatory monocytes. Alveolar macrophages were specifically impacted in response to *H. ohiense* compared to infection with H. capsulatum or mock-inoculated animals. Our findings suggested that alveolar macrophages were preferentially targeted by *H. ohiense* early after infection, despite similar rates of uptake and intracellular growth between both fungal species *in vitro*.

It is presumed that *Histoplasma* is primarily associated with alveolar macrophages after respiratory infection; however, this dogma has not been specifically examined *in vivo* or in the context of all leukocyte populations. In fact, Deepe et al. found that the majority of infected lung cells are dendritic cells (CD11c^+^ Mac-3-MHC-II^hi^ CD68^−^) 1 day after the inoculation of C57BL/6 mice with 10^6^ *Histoplasma* yeasts (20). It is important to note that Deepe et al. did not specifically analyze alveolar macrophages, and even so, less than 30% of all yeast-associated cells were Mac-3^+^ CD11c^−/lo^ macrophages at every time point analyzed (20). In that same study, both H. capsulatum (G186A) and *H. ohiense* (G217B) were primarily associated with neutrophils from 3 to 7 days after inoculation (20). It is also possible that the findings by Deepe et al. are reflective of high intranasal inocula (10^5^ to 10^6^ yeasts) compared to our experiments (2,500 yeasts), chosen because this lower inoculum equalizes lung fungal burdens of both *Histoplasma* species throughout the course of infection (19). In another study, alveolar macrophages represent only approximately 5% of all infected lung cells 7 days after mice are inoculated with 10^5^ *H. ohiense* (G217B) (37). Therefore, *H. ohiense* may productively infect alveolar macrophages relatively early after pulmonary infection; however, counter to dogma, this cell population does not appear to serve as a long-term intracellular niche for either H. capsulatum or *H. ohiense*.

Infection with *H. ohiense* was correlated with a dramatic reduction in the number of alveolar macrophages compared to either mock-infected or H. capsulatum-infected mice, and thus, we originally proposed that this was due to enhanced intracellular parasitism and subsequent cell death. There is contention as to whether *Histoplasma* induces macrophage apoptosis, and conflicting interpretations are likely a result of the technical differences among studies related to strain usage, multiplicity of infection, timing, and models of infection. *In vitro* studies have reported that cultured alveolar macrophages are apoptotic within 24 h after incubation with *Histoplasma* at a multiplicity of five yeasts per cell, and this is suggested to be due to the expression of calcium binding protein (CBP), a yeast-specific secreted effector (38, 39). Pitangui et al. examined the interaction of *Histoplasma* with AMJ2-C11 alveolar macrophages and found that apoptosis occurs within 5 h after incubation with a 5:1 ratio of yeasts to cells (40). In that study, the authors noted that microscopic imaging revealed that individual cells contain up to 24 to 30 yeasts, and thus, it is not surprising that apoptosis occurs rapidly in that scenario (40). Indeed, the multiplicity of infection has been directly correlated with cell death after *in vitro* infection (41). Deepe and Buesing showed that apoptosis of alveolar macrophages is evident 24 h after incubation, but only if a multiplicity of five or more yeasts per cell is used (41). This is in direct agreement with our *in vitro* results using AMJ2-C11 cells, as we opted to use a low multiplicity of infection to evaluate intracellular growth kinetics while avoiding overt cell death (see Fig. S5 in the supplemental material).

On the other hand, *in vivo* studies have suggested that *Histoplasma* is correlated with the inhibition of apoptosis in myeloid cell types. The peritoneal inoculation of mice with *Histoplasma* leads to a reduction in the propensity of inflammatory phagocytes to undergo apoptosis *ex vivo* (42). Likewise, macrophages represent only a minor (1%) fraction of all apoptotic lung cells, whereas T cells represent the majority (80 to 90%) of apoptotic cells on days 7, 14, and 21 after intranasal inoculation with *H. ohiense* strain G217B (43). Although a minor relative proportion of all apoptotic lung cells were Mac-1^+^ macrophages in that study, it is possible that a significant internal fraction of the macrophage population was apoptotic, but those data were not reported. Indeed, English et al. found that 3 days after inoculation with 10^5^ *H. ohiense* (G217B), approximately 20 to 30% of alveolar macrophages are apoptotic, and this was significantly reduced in mice that lacked the proapoptotic gene *CHOP* (44). It is not clear whether that result was due to direct intracellular infection *in vivo*, but it is consistent with our data suggesting that a proportion of alveolar macrophages were depleted in response to *H. ohiense*.

It is possible that the selective loss of alveolar macrophages in the lungs of *H. ohiense*-infected mice was an indirect result of the inflammatory milieu. Our findings are in agreement with Deepe et al., who posit that *Histoplasma* infection promotes a bystander effect in which uninfected macrophages undergo apoptosis, since only a minor proportion (∼10%) of apoptotic cells are directly infected (20). Bystander macrophage apoptosis was originally proposed by Kelly et al. in the context of Mycobacterium tuberculosis infection to explain how uninfected cells undergo cell death after contact with infected cells in a tumor necrosis factor alpha (TNF-α) and transforming growth factor β (TGF-β)-independent manner (45). We propose that the presence of heavily infected macrophage-like cells 8 days after *H. ohiense* infection (Fig. 3A) contributed to the loss of alveolar macrophages 12 to 16 days postinfection by either the aforementioned bystander effect or perhaps due to the elevated proinflammatory lung environment (Fig. 2F). Indeed, significantly higher concentrations of IL-1β were detected in the lungs of *H. ohiense-* but not H. capsulatum-infected mice (Fig. 7E). Moreover, the peak concentration of IL-1β in the lungs of *H. ohiense*-infected mice immediately preceded the time point when the fewest alveolar macrophages were recovered. This is consistent with the finding that IL-1β is associated with alveolar macrophage sensitization and cell death in the context of lung inflammation (46). Therefore, it is possible that the subsequent loss of alveolar macrophages was an indirect effect of the intensified rate and extent of inflammation during *H. ohiense* infection.

The outer layer of the *Histoplasma* cell wall is considered to be a primary virulence determinant of nearly all *Histoplasma* species, including H. capsulatum (15), in which α-(1,3)-glucan masks the underlying β-(1,3)-glucan from being recognized by the C-type lectin pattern recognition receptor Dectin-1 (47). In contrast, α-(1,3)-glucan is not detected in the cell wall of *H. ohiense* and is not considered to be required for virulence *in vitro* or *in vivo* in this species (48). *H. ohiense* diminishes Dectin-1-mediated recognition by the secretion of two major β-glucanases that promote virulence *in vivo* (49, 50). Despite this immune evasion strategy, the Wu-Hsieh group showed that *Histoplasma* with exposed surface β-(1,3)-glucan stimulates macrophage cytokine production via Dectin-1 (51, 52). Additionally, Dectin-2 and Dectin-1 signaling lead to pro-IL-1β expression and cathepsin B release to promote the NLRP3 inflammasome and IL-1β release (53). Indeed, IL-1β was upregulated 7 days after inoculation with 10^5^ *Histoplasma* strain 505 (53), which is an isolate with exposed β-(1,3)-glucan (52). Alveolar macrophages are known to express high levels of Dectin-1 (54), and accordingly, we observed a significantly greater activation of alveolar macrophages isolated from the lungs of mice inoculated with *H. ohiense* relative to H. capsulatum. Thus, the distinct host inflammatory patterns we observed in response to H. capsulatum and *H. ohiense* were likely attributed at least in part to differences in innate immune recognition of β-(1,3)-glucan and corresponding signaling events by mononuclear phagocytes.

The timing of *H. ohiense*-induced inflammation characterized in this study using a dose of only 2,500 yeasts was similar to studies that have used significantly higher doses. Baughman et al. reported that intranasal inoculation with 10^5^ *H. ohiense* strain G217B leads to a significant elevation of neutrophils in bronchoalveolar lavage fluid 7 days after infection relative to control mice (55), which is consistent with our results using *H. ohiense*. Cain and Deepe analyzed the progression of disease up to 14 days after inoculation with 10^6^ *H. ohiense* and showed that the percentage of Mac-1^+^ and Gr-1^+^ lung cells (which would include both neutrophils and Ly6C^hi^ monocytes) peak 7 days after infection (56). Additionally, Deepe et al. that showed *H. ohiense* infection is associated with a robust neutrophilic response relative to H. capsulatum after inoculation with 10^6^ CFU (20). Thus, we corroborated the immunological findings by Deepe et al. and demonstrated that *H. ohiense* was associated with a significantly greater neutrophilic response even in the context of similar fungal lung burdens relative to H. capsulatum using a low, sublethal inoculum.

Interestingly, in regard to fungal clearance, we observed similar numbers of yeasts localized within inflammatory foci (Fig. 3C), but significantly different fungus-associated cell types between *Histoplasma* species 16 days after inoculation (Fig. 4C). H. capsulatum was primarily associated with interstitial macrophages and Ly6C^lo^ monocytes, whereas *H. ohiense* was found with inflammatory monocytes and neutrophils (Fig. 4C). Both classically activated macrophages and neutrophils restrict the intracellular growth of *Histoplasma* (57–59). This suggests that H. capsulatum might be effectively cleared by activated tissue-resident phagocytes, while *H. ohiense* is primarily controlled by inflammatory cell types that are specifically recruited to the lung during infection. It would be interesting to investigate in future studies whether diverse *Histoplasma* species are associated with either preferential uptake or intracellular survival in specific monocyte and macrophage subsets.

The murine model of acute pulmonary histoplasmosis characterized in this study provides insight into the pathogenic strategies utilized by diverse *Histoplasma* species. The use of a low, sublethal inoculum did not elicit host inflammatory responses until at least 4 days after inoculation, despite significant fungal growth during this time period (19). This unique feature allowed us to probe the differential progression of inflammation and immune cell dynamics that occurred as a result of natural fungal proliferation in the lung, as opposed to initial inoculation with a relatively high number of yeasts. H. capsulatum G186A and *H. ohiense* G217B are the most commonly studied strains in the *Histoplasma* literature, representing the two distinct cell wall phenotypes found in all strains/species of *Histoplasma.* Therefore, these results provide a foundation for future studies to assess additional species-specific differences. Interestingly, because intranasal inoculation with this relatively low dose still resulted in systemic fungal spread (unpublished data), this model could also be utilized to investigate virulence determinants related to disseminated forms of histoplasmosis.

## MATERIALS AND METHODS

### Fungi and culture methods.

Histoplasma capsulatum (Panama/H81, G186A isolate, ATCC 26029) and Histoplasma ohiense (Nam 2, G217B isolate, ATCC 26032) were used in this study (13). The following strains that constitutively expressed *gfp* during the yeast phase were also used: H. capsulatum G186A *ura5-31 zzz*::[*P_CBP1_-gfp hph*] and *H. ohiense* G217B *ura5-41 zzz*::[*P_CBP1_-gfp hph*]. The following isogenic strains were used as negative controls: H. capsulatum G186A *ura5-31* and *H. ohiense* G217B *ura5-41*. All four of the control and *gfp*-expressing strains listed above were transformed with pCR25 (*PaURA5*) to complement uracil auxotrophy, and the methods used to generate these strains were described previously (60, 61). All strains listed above were routinely cultured at 37°C in 5% CO_2_ in *Histoplasma* macrophage medium (HMM) liquid broth or agarose plates with 0.6% agarose and 25 mM FeSO_4_ (62). To obtain dispersed yeast suspensions for *in vivo* and *in vitro* inocula, a series of low-speed centrifugation steps were performed to remove yeast aggregates, and individual yeasts were counted using a hemocytometer as described previously (19). Last, to generate fungal growth curves in mammalian cell culture media, log-phase 5-ml HMM broth cultures were back-diluted into 25-ml complete Dulbecco modified Eagle medium (DMEM) with l-cystine after being standardized to a starting optical density at 600 nm (OD_600_) of 0.05 and incubated in an orbital shaker at 120 rpm at 37°C with CO_2_. Optical densities were recorded every 24 h.

### Murine model of acute pulmonary histoplasmosis.

Male C57BL/6J mice (4 to 6 weeks old; Jackson Laboratory) were sedated using a combination of ketamine and xylazine before being inoculated intranasally with 20 μl of *Histoplasma* yeasts (2.5 × 10^3^ CFU) in HMM, which was described previously as a low, sublethal dose (19). Mock-infected mice were inoculated with 20 μl of HMM. At various time points after inoculation, mice were euthanized with a lethal dose of sodium pentobarbital and the whole lung was collected for histology, digested into a single-cell suspension, or homogenized for cytokine analyses. These studies were approved by the University of North Carolina Office of Animal Care & Use.

### Tissue processing.

The lung was perfused by injection of 10 ml cold phosphate-buffered saline (PBS) into the right ventricle of the heart prior to being collected for either histology or flow cytometry. For histology, lungs were inflated with 10% buffered formalin solution by cannulation of the trachea and then incubated for at least 48 h before being washed with PBS and embedded in paraffin. Three 5-μm-thick lung sections separated by 200-μm skips were collected per mouse and were subjected to either hematoxylin and eosin (H&E) staining or immunohistochemistry.

To generate single-cell suspensions for flow cytometry, the lung was enzymatically digested using an approach modified from Yu et al. to improve cell yields and avoid cell loss/damage associated with mechanical dissociation (24), which was described previously in detail involving collagenase and dispase (63). Briefly, lungs were inflated via tracheal cannulation with 1 ml digestion buffer, the trachea was closed with a suture, and then the whole lung was incubated for 45 min at 37°C before being vortexed in a 30-ml total volume to dissociate the partially digested tissue into a single-cell suspension (63). Next, lung cells were subjected to red blood cell lysis using ACK buffer, washed with cold flow cytometry staining buffer (see recipe below) by centrifugation (300 × *g* for 6 min), filtered through a 70-μm cell strainer, and then kept on ice.

### Histopathology.

The workflow used to quantify the size and number of consolidated lung regions is described in Fig. S1. Bright-field images of H&E-stained lung sections were acquired using a 4× Olympus Plan Fluorite objective (0.13 numerical aperture) in an overlapping, tile-scanning manner, and the resulting fields were merged automatically in Adobe Photoshop using the Photomerge tool. Consolidated airways and inflammatory foci were traced in a semi-unbiased manner using the magic wand tool, which preferentially outlined the area of each lesion based on pixel tone and color relative to normal lung tissue. ImageJ was used to quantify whole-lung area using the traditional threshold tool, whereas the area of traced inflammatory foci was determined using the color threshold tool after filling in the outlined individual foci. The proportion of each lung section associated with inflamed regions was calculated by dividing the combined area of the inflamed regions by the total lung area. A single 4× field from one of the six tissue sections analyzed per group was previously published as a descriptive, representative image (19).

### Immunohistochemistry.

Five-micron-thick lung sections were deparaffinized using xylene followed by a graded alcohol-based series of rehydration steps (100% ethanol [EtOH], 95% EtOH, and 80% EtOH) followed by distilled water. Antigen retrieval was performed by boiling slides in citrate-based solution (10 mM sodium citrate, 0.05% Tween 20 [pH 6.0]) for 20 min. Slides were washed in TBS-T (Tris-buffered saline with 0.1% Tween 20), blocked using normal goat serum from a Vectastain ABC rabbit IgG kit (Vector Laboratories) for 30 min at room temperature, and then washed again in TBS-T. Next, slides were incubated with the primary antibody (rabbit anti-GFP [EPR14104], Abcam, 1:100 dilution) at 4°C overnight in a humidity chamber. The next morning, slides were washed multiple times in TBS-T, and endogenous peroxidase activity was blocked using 3% hydrogen peroxide in water for 10 min at room temperature. After additional washes with TBS-T, the primary antibody was detected using SignalStain Boost IHC Detection Reagent HRP Rabbit (catalog no. 8114; Cell Signaling Technology) for 30 min at room temperature. Multiple washes with TBS-T were performed prior to incubating sections in freshly prepared diaminobenzidine (DAB) substrate (ab64238, Abcam) for 2 min followed by additional wash steps, and then hematoxylin counterstaining for 90 s. Slides were dehydrated using a graded alcohol series (50% EtOH, 70% EtOH, and 100% EtOH) followed by xylene, and Permount (Fisher Scientific) was used to mount glass coverslips. Slides were scanned using the 40× objective of an Aperio Versa 200 (Leica Biosystems) pathology scanner in the Translational Pathology Laboratory in the University of North Caroling (UNC) School of Medicine. Aperio ImageScope v12 software was used to quantify yeasts with the counting tool, and the pen tool was used to measure the area of inflammatory lesions.

### Flow cytometry of lung cells.

Three milliliters (∼1 × 10^6^ cells) of each lung single-cell suspension (10% of total recovered cells) was subjected to fluorescence-conjugated antibody staining. Cells were washed in flow cytometry staining buffer (Ca^2+^/Mg^2+^-free PBS containing 5% fetal bovine serum [FBS] and 2 mM EDTA) and then incubated with a fixable live/dead viability dye (Zombie Red; BioLegend) for 15 min at room temperature. Next, cells were washed with cold buffer and Fc receptors were blocked by incubation with anti-mouse CD16/32 (clone 93, BioLegend) on ice for 5 min, followed by a 30-min incubation on ice with a cocktail of titrated, primary-conjugated fluorescent antibodies from BioLegend (fluorescein isothiocyanate [FITC]-Ly6C [HK1.4], Brilliant Violet 510-Ly6G [1A8], phycoerythrin-CD11c [N418], allophycocyanin-CD11b [M1/70], Brilliant Violet 711-I-A/I-E [M5/114.15.2], Brilliant Violet 421-CD64 [X54-5/7.1]) and the following from Invitrogen; allophycocyanin-eFluor780-CD45 (30-F11), peridinin chlorophyll protein (PerCP)-eFluor 710-Siglec F (1RNM44N), and phycoerythrin-Cyanine7-CD24 (M1/69). For *gfp-*expressing yeast studies, the following antibodies from BioLegend were used to account for the detection of GFP in the FITC channel; phycoerythrin-Ly6C (HK1.4) and Brilliant Violet 605-CD11c (N418). Last, cells were washed twice with cold staining buffer, fixed with 0.5 ml of 2% buffered formalin for 20 min, resuspended in 250 μl staining buffer, and acquired using a Thermo Fisher Attune NxT cytometer. All flow cytometry files were analyzed using FlowJo software v10.

The panel of antibodies listed above was derived from multiple schemes to phenotype immune cell populations with a focus on the myeloid compartment in both healthy and inflamed lungs of mice (23, 24). The workflow used to discriminate distinct cell populations using the *t*-distributed stochastic neighbor embedding (tSNE) algorithm in FlowJo software is described in Fig. S2A. Manual gating was performed to exclude doublets/aggregates followed by gating live CD45^+^ lung cells, which were downsampled to an equal number of events per sample before concatenating all files together at each time point (Fig. S2A). The unsupervised tSNE algorithm created two new derived parameters based on expression patterns of the surface antigens listed above, and after optimizing input parameters (iterations, 800; perplexity, 30), all events were reduced into one two-dimensional space to reveal uniquely clustered events. To identify and confirm the identity of the clusters that were segregated in an unbiased manner, we used a sequential gating scheme to phenotype all CD45^+^ cells (Fig. S2B), as well as the tSNE heat maps that displayed the relative expression levels of each antigen (Fig. S2C). To visualize all cell subsets within the two tSNE-derived parameters, we overlaid the gated populations onto the tSNE plots and then backgated to compare experimental groups. In the manual gating scheme, fluorescence-minus-one samples were employed as negative gating controls, particularly with CD11c, CD64, major histocompatibility complex class II (MHC-II), and CD24. To determine which lung cells were associated with *gfp-*expressing yeast, lung cells isolated from mice inoculated with control *Histoplasma* strains were used to define the threshold of GFP positivity for each cell population.

### Quantitative imaging flow cytometry of infected AMJ2-C11.

The murine alveolar macrophage cell line AMJ2-C11 was purchased from ATCC and cultured in DMEM (Gibco) with 4.5 g/liter d-glucose, 110 mg/liter sodium pyruvate, l-glutamine, 5 mM HEPES, 5% FBS (HyClone), in addition to 84 mg/liter l-cystine to support fungal growth. For infection assays, the wells of 24-well plates were seeded with 2.5 × 10^5^ cells per well in 1 ml media for infection assays and incubated at least 1 h prior to inoculation with 1.25 × 10^5^ to 2.5 × 10^5^ *Histoplasma* yeasts in 50 μl HMM. One hour after infection, extracellular yeasts were removed using prewarmed PBS and three wash steps in a swinging-bucket centrifuge rotor at 300 × *g* for 5 min each. Cells were cultured in 1 ml complete DMEM for 1 to 3 days, with half of the media being replaced after 48 h for the 72-h time point. At each time point, cells were gently removed using prewarmed PBS with 0.6 mM EDTA and pooled into 5-ml round-bottom tubes prior to being incubated with a fixable live/dead viability dye (Zombie NIR; BioLegend) for 15 min at room temperature. Last, cells were washed twice with cold staining buffer, fixed with 0.5 ml 2% buffered formalin for 20 min, and then transferred into 1.5-ml microcentrifuge tubes prior to being analyzed using an Amnis ImageStreamX Mark II. Ten thousand focused events were acquired per sample using the 40× objective along with the following channels: 1, bright-field; 2, *gfp-*expressing yeasts; 6, side scatter (SSC); 9, bright-field; 12, viability dye. Negative gating thresholds were determined using uninfected AMJ2-C11 cells, and the channel 2 laser intensity was set using yeasts alone. Using Amnis IDEAS application v6.2, we first confirmed that the GFP signal associated with each cell was intracellular and not surface bound, which was reflected by a positive internalization score (typically a median of 4 to 5). All files were acquired with the same target intensities and batched using the same mask features and truth populations in the Spot Count feature to standardize the quantification of intracellular yeasts.

### Cytokine analysis.

The whole lung was collected in a 50-ml conical tube, homogenized in 5 ml cold HMM, and then immediately spiked with protease inhibitor cocktail (1:100, Calbiochem) after being transferred to microcentrifuge tubes. Samples were centrifuged at 16,000 × *g* for 20 min at 4°C, and supernatants were stored at –80°C prior to analysis. Supernatants were thawed on ice and diluted twofold in assay buffer for use in multianalyte flow cytometry-based LEGENDplex kits (BioLegend) in a V-bottom 96-well plate. Data were acquired and analyzed according to the manufacturer’s instructions.

### Statistical analyses.

Statistical inferences were determined using analyses in GraphPad Prism version 8.3. Specific tests that were performed are listed in the figure legend associated with each data set along with the *P* value designation.
