# Supplementary figures and images for: Biodiverse Histoplasma Species Elicit Distinct Patterns of Pulmonary Inflammation following Sublethal Infection
Source: mSphere. 2020 Aug 26;5(4):e00742-20. doi: 10.1128/mSphere.00742-20 (PMC7449625; doi:10.1128/mSphere.00742-20)

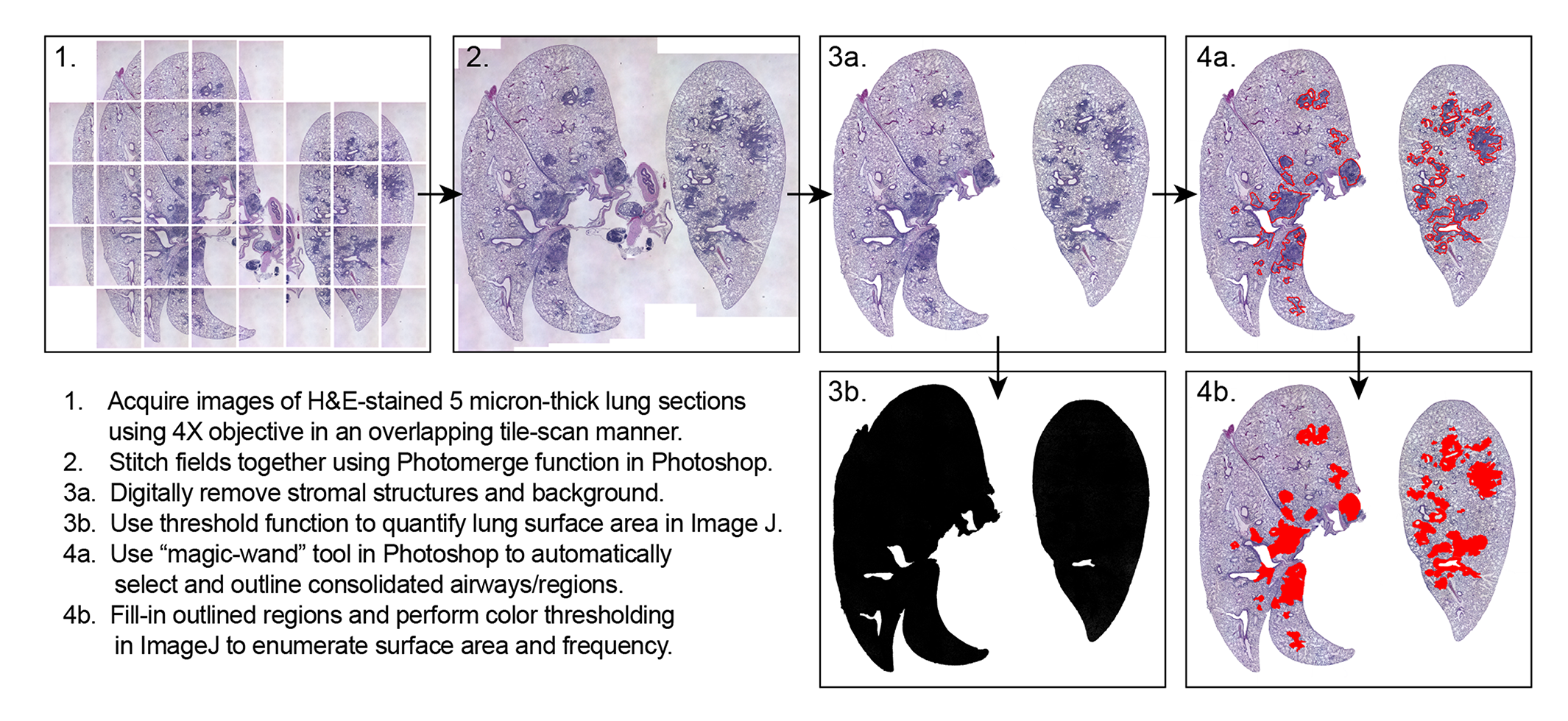

Supplement: FIG S1 [file mSphere.00742-20-sf001.tif]

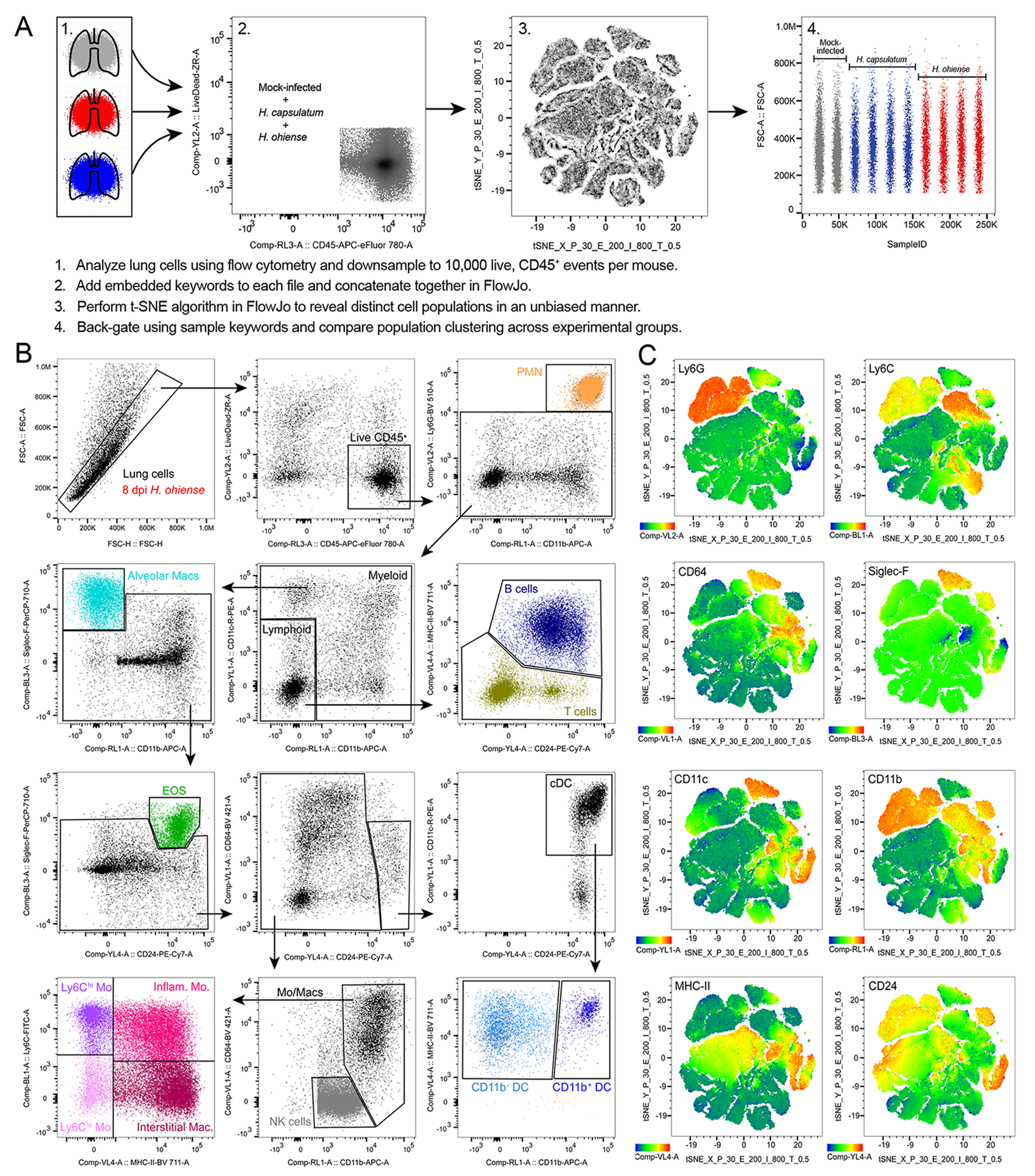

Supplement: FIG S2 [file mSphere.00742-20-sf002.tif]

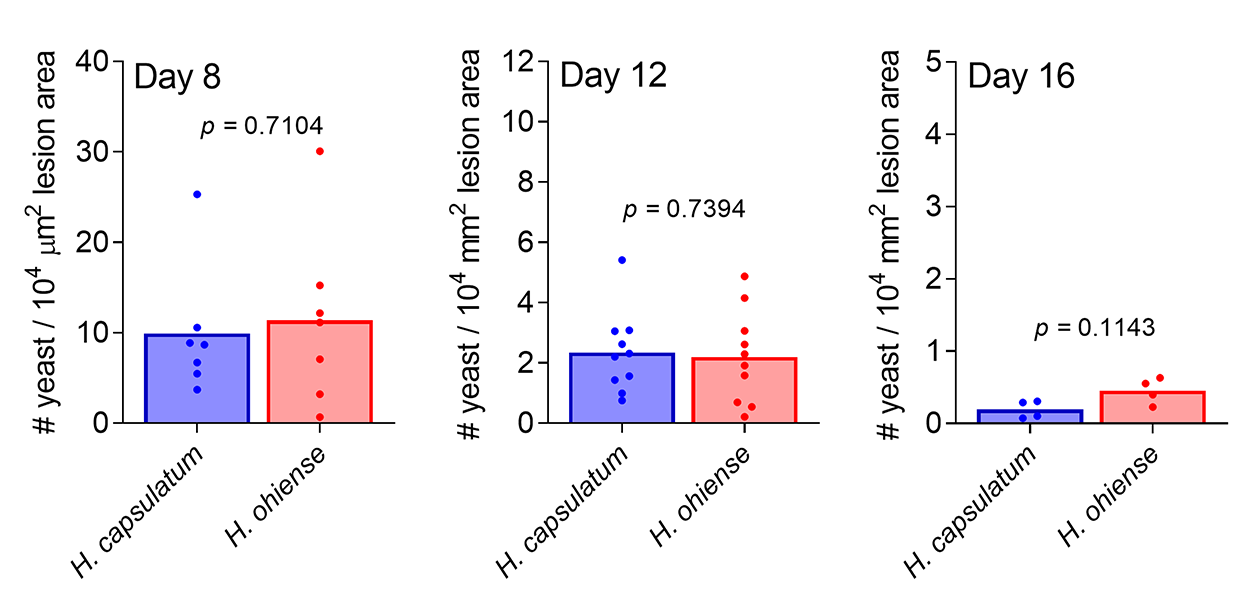

Supplement: FIG S3 [file mSphere.00742-20-sf003.tif]

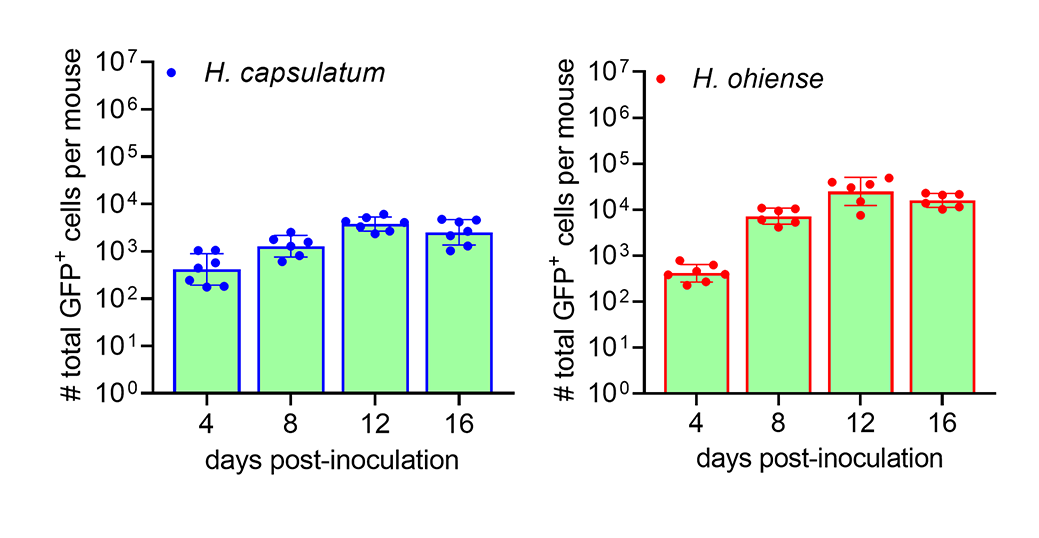

Supplement: FIG S4 [file mSphere.00742-20-sf004.tif]

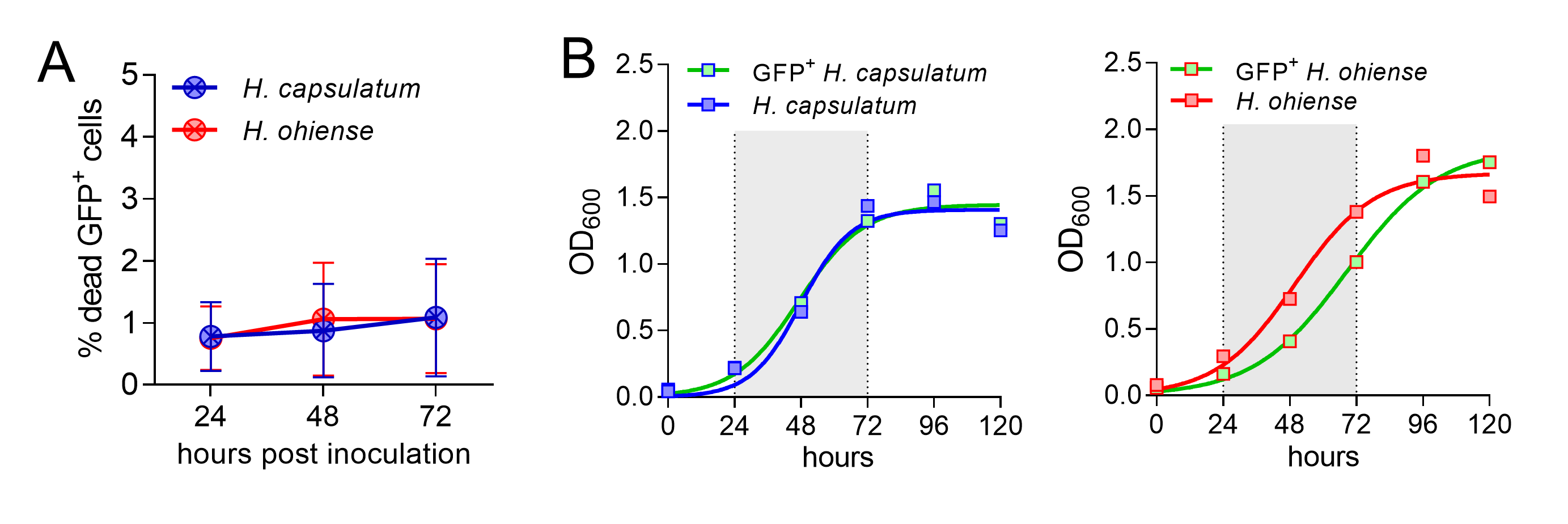

Supplement: FIG S5 [file mSphere.00742-20-sf005.tif]
